# Editorial: Application of plant secondary metabolites to pain neuromodulation, volume III

**DOI:** 10.3389/fphar.2023.1166272

**Published:** 2023-02-21

**Authors:** Rajeev K. Singla, Adriana Gibara Guimarães, Gokhan Zengin

**Affiliations:** ^1^ Institutes for Systems Genetics, Frontiers Science Center for Disease-Related Molecular Network, West China Hospital, Sichuan University, Chengdu, China; ^2^ iGlobal Research and Publishing Foundation, New Delhi, India; ^3^ Federal University of Sergipe, São Cristóvão, Brazil; ^4^ Selçuk University, Konya, Türkiye

**Keywords:** neuropathic pain, nociception, anti-inflammatory agents, phytochemicals, polyphenols, pain

Pain is a distressing condition that is often associated with various ailments, including metabolic disorders, *viz.* cancer and diabetes, neurological diseases like Parkinson’s disease, and chronic infectious diseases (Li et al., 2019; Yang, 2019; Singla et al., 2021a; Singla et al., 2021b; Singla et al., 2022b). Electrical interface-based neuromodulation has shown promise in the treatment chronic pain, significantly where high attrition rates, costs, or regulatory restrictions limit pharmacological agents. To ensure the safe and appropriate use of neurostimulation techniques, the practice guidelines developed by organizations such as the International Neuromodulation Society and the Indian Society for Study of Pain (Deer et al., 2014a; Deer et al., 2014b; James et al., 2018; Thota et al., 2020; Singla et al., 2022b).

In addition to traditional pharmacological and neurostimulator approaches, natural products have emerged as a potential avenue for pain relief (Scotti et al., 2016; Dangar and Patel, 2021; Singla et al., 2021b; Swarnkar et al., 2021; Garg et al., 2022; Kumar et al., 2022; Rauf et al., 2022; Singla et al., 2022a; Singla et al., 2023). Nature has provided various molecules, such as capsaicin, resiniferatoxin, morphine, lipoxin A4, and cannabidiol, which have significantly alleviated pain (Jin et al., 2020; Singla et al., 2020; Singla et al., 2021b). Natural products, in the form of plants, animals, and microbes, has always served as a goldmine and sustainable resource for the production of various compounds that can effectively alleviate hallmark traits of various diseases and disorders (Ramesha et al., 2011; Singla, 2014; Singla and Dubey, 2019; Singla et al., 2021c). Nanotechnological approaches can further enhance the therapeutic properties of pain-related medications by increasing their bioavailability, ADME properties, and site-specific actions (Yetisgin et al., 2020; Ansari and Goomer, 2022; Patil et al., 2022). Recent research has explored the clinical translational potential of gold nanoparticles as an effective neuro-medicine (Mishra et al., 2022). By scientifically exploring the rational use of novel formulation strategies in natural product-based studies, such research may lead to the development of alternative or complementary treatment methods for pain management (Jayasawal et al., 2022; Patel et al., 2022). Thus, this Research Topic aims to collect articles investigating plant metabolites’ potential for pain neuromodulation to provide additional insights in this direction.

Cheng Xu and the team published their clinical trial-based article entitled “The median effective analgesic concentration of ropivacaine in ultrasound-guided interscalene brachial plexus block after arthroscopic rotator cuff repair.” Cheng Xu and the team performed this study on 40 arthroscopic rotator cuff repair (ARCR) patients. They evaluated the mean effective analgesic concentration (MEAC) when treating ARCR patients with 10 mL of ropivacaine. They also assessed sufentanil consumption, the onset time of sensory block and motor block, and some other parameters.

Liqiong Yu and the team summarized the studies on traditional Tibetan medicine and published a review article entitled “Traditional Tibetan medicine: therapeutic potential in rheumatoid arthritis.” In the manuscript, they analysed the common pathways regulating the aberrant pathophysiology in rheumatoid arthritis. They have also made a comparative analysis between the 27 species that were documented as traditional Tibetan medicines and had the potential to manage rheumatoid arthritis cases. The data gathered from various Tibetan medicine monographs and online Chinese and international databases.

Yuan Kang and the team published their research article entitled “Anti-oxidative and anti-inflammatory activities of the ethanol extract of edible flower from Chimonanthus praecox.” *Chimonanthi Praecocis* Flos is commonly known as the wintersweet flower, and it is an edible flower. The ethanolic extract obtained from these flower buds was subjected to HPLC for component analysis. To validate the anti-oxidative and anti-inflammatory activities, they have performed varied *in vitro* and *in vivo* studies, especially that related to the measurement of intracellular reactive oxygen species (ROS), intracellular NADPH oxidase, mtROS, supernatant pro-inflammatory mediators, iNOS, NLRP3 inflammasome activation, luciferase reporter gene, and mouse endotoxemia model.

Reshmi Akter and the team published their research article entitled “Pomegranate juice fermented by tannin acyl hydrolase and Lactobacillus vespulae DCY75 enhance estrogen receptor expression and anti-inflammatory effect.” With the aid of tannin acyl hydrolase and *Lactobacillus vespulae*, they transformed hydrolyzable tannins present in pomegranate juice into ellagic acid. Fermented pomegranate juice was thus enriched in ellagic acid. They conducted various *in vitro* experiments to validate the upregulation of estrogen receptor expression as well as anti-inflammatory potential.

Keun-Tae Park and the team published their research article entitled “The involvement of the noradrenergic system in the antinociceptive effect of cucurbitacin D on mice with paclitaxel-induced neuropathic pain.” Paclitaxel, a widely known anticancer drug, is also well known for its inducing effect as peripheral neuropathy. They studied the alleviating effects of cucurbitacins B and D on paclitaxel-induced neuropathic pain in mice. But cucurbitacins B expressed higher cytotoxicity for the non-cancerous cell line, which resulted in removing this molecule from further experiments. Without affecting the anticancer potential of paclitaxel, cucurbitacin D expressed potential in reducing neuropathic pain, and authors have validated it with a series of experiments and explored the mechanism of action of cucurbitacin D in peripheral neuropathy.

This Research Topic thus covered one clinical trial, one review, and three original research articles. This Research Topic offers a comprehensive overview of the potential of natural products in managing various forms of neuropathic pain and neuroinflammation. Further translational studies are necessary to ensure the clinical applicability of these natural products. Such studies may shed light on the safety, efficacy, and optimal dosage of these natural products, as well as possible drug-drug interactions. This will help advance the development of alternative or complementary therapies to manage pain.
